# Association between childhood tonsillectomy and increased risk of autoimmune disease: a nationwide population-based cohort study

**DOI:** 10.3389/fmed.2026.1892240

**Published:** 2026-08-11

**Authors:** Young-Soo Chang, Eun-Jung Park, Chan Hong Jeon, Hyemin Jeong

**Affiliations:** 1 Department of Otorhinolaryngology-Head and Neck Surgery, Sanggye Paik Hospital, College of Medicine, Inje University, Seoul, Republic of Korea; 2 Department of Internal Medicine, Division of Rheumatology, National Medical Center, Seoul, Republic of Korea; 3 Department of Internal Medicine, Division of Rheumatology, Soonchunhyang University Bucheon Hospital, Soonchunhyang University College of Medicine, Bucheon, Republic of Korea

**Keywords:** adenoidectomy, autoimmune disease, childhood, cohort study, tonsillectomy

## Abstract

**Objectives:**

Tonsils and adenoids play crucial roles in the immune system, particularly during early childhood. This study investigated the association between childhood tonsillectomy and/or adenoidectomy and the subsequent development of autoimmune disease.

**Methods:**

Using nationwide claims data from the Health Insurance Review and Assessment Service, we identified children aged 0–12 years who received medical care for tonsillar or adenoid disease between January 2007 and January 2008. Participants were classified into surgery (tonsillectomy or adenoidectomy between 2007 and 2013) and non-surgery groups and followed until autoimmune disease diagnosis or June 2023. A Cox proportional hazards model was used to analyze the risk of developing autoimmune disease according to surgery.

**Results:**

A total of 84,030 children in the surgery group and 336,120 age- and sex-matched children in the non-surgery group were analyzed, with a mean follow-up duration of 15.56 years. The incidence rates of autoimmune diseases in the non-surgery and surgery groups were 65.3 and 86.5 per 100,000 person-years, respectively. Surgery (vs. non-surgery) was associated with an increased risk of developing systemic lupus erythematosus [hazard ratio (HR) 2.20, 95% confidence interval (CI) 1.55–3.13], ankylosing spondylitis (HR 1.35, 95% CI 1.06–1.72), juvenile idiopathic arthritis (HR 1.69, 95% CI 1.19–2.39), type 1 diabetes (HR 1.34, 95% CI 1.14–1.58), Hashimoto's thyroiditis (HR 1.39, 95% CI 1.22–1.57), and Graves' disease (HR 1.38, 95% CI 1.22–1.56). Overall, surgery was associated with an increased risk of developing autoimmune disease (HR 1.32, 95% CI 1.23–1.41).

**Conclusion:**

Tonsillectomy and/or adenoidectomy during childhood was associated with an increased risk of developing autoimmune diseases later in life. These findings suggest a possible association between tonsillar and adenoid immune function and later autoimmune disease risk.

## Introduction

Tonsils and adenoids constitute the majority of Waldeyer's ring. This ring of tissue contributes to the body's immune defense by providing protection against infections that enter through oral and nasal cavities. As secondary lymphoid organs, tonsils and adenoids are particularly vital components of the immune system during early childhood (1). Tonsils and adenoids contribute to the generation of memory B cells and immunoglobulin A (IgA)-producing effector B lymphocytes, and they harbor a range of immune cell populations, including B cells, T cells, dendritic cells, and macrophages, that support immune surveillance (2). Recent evidence further supports the immunological role of Waldeyer's ring. Ramirez et al. demonstrated the presence of antigen-specific memory B and T cells in adjacent upper airway tissues, highlighting their contribution to mucosal immune memory and local immune surveillance (3). These organs are present at birth, but are relatively small at that point. They exhibit rapid growth and reach their maximum sizes between the ages of 6 and 8 years (4). Involution of the lymphoid tissue begins around puberty, and little lymphoid tissue is left in old age (5). Tonsillectomy, a commonly performed surgical procedure in pediatric populations, is generally indicated for recurrent tonsillitis, chronic tonsillitis, tonsillar abscess, tonsillar hypertrophy, and sleep apnea (6).

It is generally accepted that the effect of tonsillectomy on immune function is minimal and not clinically significant. One meta-analysis found no clinically significant adverse effects of tonsillectomy on the immune system (7). However, the long-term impacts of tonsillectomy in childhood on the subsequent development of autoimmune diseases remain unclear. Studies of the relationship between tonsillectomy and rheumatoid arthritis (RA) have yielded conflicting results. Some suggest that tonsillectomy increases the risk of developing RA (8, 9), while others have found no significant difference in tonsillectomy rates between RA patients and controls (10, 11). Tonsillectomy reportedly increases the risk of developing Crohn's disease while no association has been found between tonsillectomy and ulcerative colitis (12). One notable and comprehensive nationwide study in Sweden that examined the incidence of autoimmune diseases subsequent to tonsillectomy found an association between the surgical procedures and an elevated risk of developing autoimmune diseases in the future (13). That study involved large-scale research, but is the only one of its kind, while others have small sample sizes and case-control designs, limiting our ability to interpret the results.

This study aimed to investigate the association between childhood tonsillectomy and the subsequent development of autoimmune disease using nationwide claims data.

## Methods

### Data resource

We used data from the Health Insurance Review and Assessment Service (HIRA) of South Korea. South Korea's mandatory single-payer, universal-coverage National Health Insurance System (NHIS) has covered approximately 98% of the country's population since 2000. The HIRA assesses medical service fees, evaluates the quality of healthcare, and ensures the appropriateness of medical services. The HIRA database is a comprehensive national registry encompassing the South Korean population. This extensive database contains a wide array of information, including demographic data, medication records, medical procedures, and diagnostic codes based on the International Classification of Diseases, Tenth Revision (ICD-10). HIRA data also contain rare intractable disease (RID) codes. RID codes are assigned following a comprehensive evaluation by the treating physician and the NHIS to meet standardized diagnostic criteria (14). Registration with an RID code qualifies patients to receive coverage for medical expenses and medication costs through the NHIS.

### Study population

The study included individuals aged 0 to 12 years who received medical treatment for tonsil and adenoid diseases (J03, J030, J0300, J0301, J038, J0380, J0381, J039, J0390, J0391, J35, J350, J351, J353, J358, J359, and J36) between January 1, 2007, and January 1, 2008. Individuals diagnosed with malignant diseases (any C code) or benign neoplasms of the brain (D330, D331, D332, D430, D431, and D432) at any time were excluded from the study. Individuals diagnosed with autoimmune diseases before the index date (the date of medical treatment for tonsil or adenoid diseases) were excluded. Additionally, those diagnosed with autoimmune diseases either before or within 6 months after tonsil or adenoid surgery were also excluded.

The study population was divided into two groups: a surgical group of individuals who underwent tonsillectomy or adenoidectomy between January 2007 and May 2013, and a non-surgical group of those who did not undergo surgery. Individuals who underwent tonsil or adenoid surgery between June 2013 and June 2023 were excluded. Surgery was defined as having at least one of the following procedure codes from the HIRA: Q2280 (adenoidectomy); Q2281 (endoscopic adenoidectomy); or Q2300 (tonsillectomy). The indication for surgery was categorized as either non-inflammatory or inflammatory conditions. Non-inflammatory conditions include enlargement or other chronic alterations of the tonsils and adenoids, such as tonsillar hypertrophy, adenoidal hypertrophy, and other chronic pathologies affecting these structures. Inflammation covers both acute and chronic infections, including abscesses of the tonsils or adenoids.

This study adhered to the Declaration of Helsinki, and the study protocol was reviewed and approved by the Institutional Review Board (IRB) of Soonchunhyang University Hospital (IRB No. 2024-01-011). Informed consent from participants was waived because the analysis used anonymous and public data.

### Follow-up and study outcomes

The endpoint of the study was the occurrence of newly diagnosed autoimmune diseases. Participants were followed from the index date (the date of medical care for tonsil or adenoid diseases between January 2007 and January 2008) until either a diagnosis of an autoimmune disease or the study's end date (June 2023).

Disease categories were defined according to ICD-10 and RID codes available in the HIRA database. The development of an autoimmune disease was defined as a case in which the ICD-10 code (as a primary or secondary diagnosis) and the corresponding RID code for each disease were both registered. Only incident cases after the index date were included in the analysis. The ICD-10 code and the corresponding RID codes used to identify 22 autoimmune diseases are listed in Supplementary Table 1. The definition of inflammatory myositis includes polymyositis and dermatomyositis. The definition of antineutrophil cytoplasmic antibody–associated vasculitis includes Wegener's granulomatosis, microscopic polyangiitis, and Churg–Strauss syndrome. For consistency with current pediatric rheumatology nomenclature, juvenile rheumatoid arthritis (ICD-10 code M080) is referred to as juvenile idiopathic arthritis (JIA) throughout this study. JIA-related categories (M080, M082, and M083) were analyzed separately according to the corresponding ICD-10 administrative codes available in the HIRA database. Among these 22 autoimmune diseases, three (type 1 diabetes mellitus [DM], Hashimoto's thyroiditis, and Graves' disease) are not classified as an RID and were therefore defined exclusively by their ICD-10 codes. Autoimmune disease 1 refers to the sum of all individual diseases, with cases involving two or more diseases counted as one person. Autoimmune disease 2 refers to the sum of individual diseases, excluding type 1 DM, Hashimoto's thyroiditis, and Graves' disease, with cases involving multiple diseases still counted as one person. This study investigated 22 systemic autoimmune and immune-mediated inflammatory diseases. Although some conditions, such as Behçet's disease, polyarteritis nodosa, and Takayasu arteritis, are currently considered immune-mediated or autoinflammatory rather than classical autoimmune diseases, they were included because they are immune-mediated inflammatory disorders (15, 16). For simplicity, the term “autoimmune diseases” is used throughout the manuscript as an umbrella term encompassing systemic autoimmune and immune-mediated inflammatory diseases.

### Statistical analysis

The characteristics of the study cohort were summarized using descriptive statistics. Continuous variables were reported as the mean with standard deviation, while categorical variables were expressed as numbers and percentages. The incidence rates of each outcome variables were calculated by dividing the number of incident events by the total follow-up period (100,000 person-years). Hazard ratios (HRs) and 95% confidence intervals (CIs) were estimated using Cox proportional hazards regression in the age- and sex-matched cohort. Age and sex were controlled through the matching process, and no additional covariates were included in the Cox regression model. Kaplan–Meier curves were used to compare the cumulative incidence of autoimmune diseases between the non-surgery and surgery groups. A subgroup analysis was conducted for the surgery group. A *p*-value of less than 0.05 was considered statistically significant. Statistical analysis was performed using SAS (version 9·4; SAS Institute, Cary, NC, USA) and R (version 4·2·3; R Foundation for Statistical Computing, Vienna, Austria) software.

## Results

### Baseline characteristics of the study population

**Figure 1:**
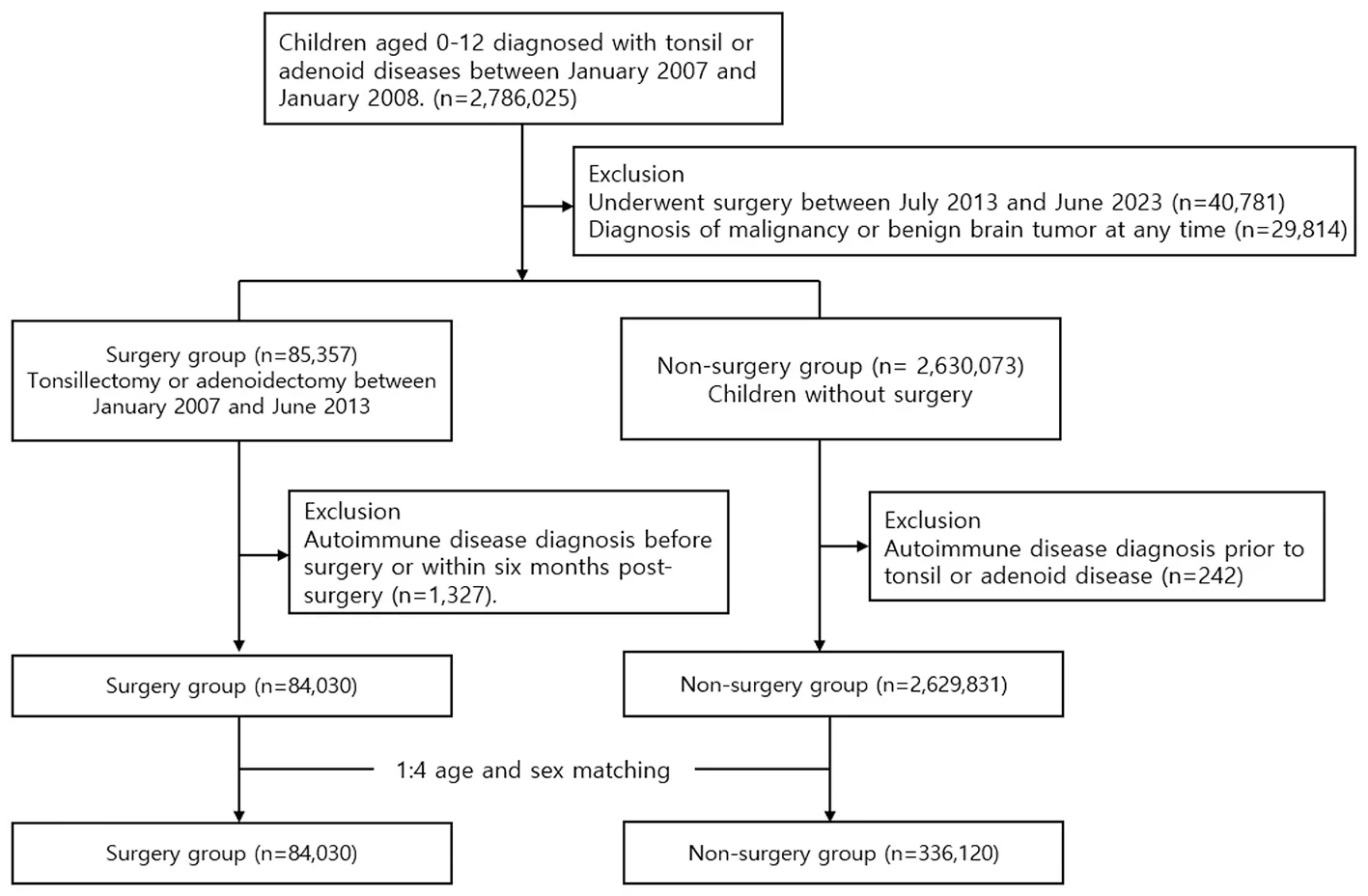
Flowchart of the study population.

Among 2,786,025 children aged 0 to 12 years diagnosed with tonsil or adenoid diseases between January 2007 and January 2008, 40 781 who underwent tonsillectomy and/or adenoidectomy between June 2013 and June 2023 were excluded. Children who underwent tonsillectomy or adenoidectomy between January 2007 and May 2013 were classified as the surgery group (*n* = 85,357), while those without a history of surgery were classified as the non-surgery group (*n* = 2,630,073). After applying the additional exclusion criteria, the final surgery group comprised 84,030 participants, and 336,120 were selected from the non-surgery group through 1:4 age- and sex-matching. A flowchart of the study population selection is shown in Figure 1.

Table 1 presents the baseline characteristics of the study population at the index date. The mean age of participants 5.25 years (SD 3.17), and 62.1% were male. The most common diagnosis was acute tonsillitis.

**Table 1:** Baseline characteristics at the time of tonsil or adenoid disease between 2007 and 2008.

Variable	Overall (*n* = 420,150)	Surgery (*n* = 84,030)	Non-surgery (*n* = 336,120)	*p*
Age, years	5.25 (3.17)	5.25 (3.17)	5.25 (3.17)	1.000
Sex				1.000
Male	260,765 (62.1%)	52,153 (62.1%)	208,612 (62.1%)	
Female	159,385 (37.9%)	31,877 (37.9%)	127,508 (37.9%)	
Disease				< 0.001
Acute tonsillitis	366,145 (87.1%)	60,487 (72.0%)	305,658 (90.9%)	
Chronic tonsillitis	8,239 (2.0%)	4,137 (4.9%)	4,102 (1.2%)	
Peritonsillar abscess	26,076 (6.2%)	4,339 (5.2%)	21,737 (6.5%)	
Non-inflammation^*^	19,690 (4.7%)	15,067 (17.9%)	4,623 (1.4%)	

Data are mean (SD) or *n* (%). ^*^Non-inflammation included tonsillar hypertrophy, adenoidal hypertrophy, and other chronic non-infectious tonsillar or adenoidal conditions.

### Incidence of autoimmune diseases between the surgery and non-surgery groups

The overall mean follow-up duration was 15.56 years: 15.53 years for the non-surgery group and 15.67 years for the surgery group. Supplementary Table 2 summarizes the incidence of autoimmune diseases during follow-up. The surgery group showed significantly higher incidences of systemic lupus erythematosus (SLE), ankylosing spondylitis (AS), JIA, and endocrine autoimmune diseases, including type 1 DM, Hashimoto's thyroiditis, and Graves' disease, than the non-surgery group. Overall, the incidence of autoimmune diseases remained significantly higher in the surgery group, even after excluding the three endocrine diseases defined only by ICD-10 codes.

### Incidence rates of autoimmune diseases in surgery and non-surgery groups

Table 2 presents the incidence rates of autoimmune diseases in the surgery and non-surgery groups. The overall incidence rates of all autoimmune diseases in the non-surgery and surgery groups were 65.26 and 86.50 per 100,000 person-years, respectively, with an incidence rate ratio (IRR) of 1.33 (95% CI 1.24–1.42; *p* < 0.001). These association remained significant after excluding the three endocrine diseases defined only by ICD-10 codes (IRR 1.24, 95% CI 1.10–1.40; *p* < 0.001). The incidence rate of autoimmune diseases was higher in females than in males. In both sexes, the incidence rate was higher in the surgery group than in the non-surgery group. The IRR was 1.26 (95% CI 1.14–1.39; *p* < 0.001) in males and 1.38 (95% CI 1.26–1.51; *p* < 0.001) in females.

**Table 2:** Comparison of the incidence rate of autoimmune diseases between the surgical and non-surgical groups during the follow-up period (2007–2023).

	All, incidence rate per 100,000 person-years (95% CI)			Male, incidence rate per 100,000 person-years (95% CI)			Female, incidence rate per 100,000 person-years (95% CI)		
Variable	Overall (*n* = 420,150)	Non-surgery (*n =* 336,120)	Surgery (*n =* 84,030)	Overall (*n =* 260,765)	Non-surgery (*n =* 208,612)	Surgery (*n =* 52,153)	Overall (*n =* 159,385)	Non-surgery (*n =* 127,508)	Surgery (*n =* 31,877)
Rheumatoid arthritis	0.54 (0.37–1.49)	0.52 (0.34–1.51)	0.61 (0.26–2.39)	0.25 (0.12–0.91)	0.25 (0.11–0.98)	0.25 (0.03–1.77)	1.00 (0.65–2.96)	0.95 (0.57–2.98)	1.20 (0.44–5.20)
Systemic lupus erythematosus	2.08 (1.75–4.92)	1.67 (1.33–4.11)	3.72 (2.75–9.84)	0.72 (0.48–2.06)	0.56 (0.33–1.76)	1.35 (0.67–4.83)	4.29 (3.52–10.37)	3.46 (2.70–8.77)	7.57 (5.36–20.79)
Ankylosing spondylitis	5.34 (4.79–11.86)	4.96 (4.38–11.21)	6.84 (5.50–16.80)	7.39 (6.58–16.56)	7.00 (6.12–15.95)	8.96 (7.02–22.53)	2.01 (1.49–5.29)	1.66 (1.14–4.65)	3.39 (1.97–10.85)
Systemic sclerosis	0.18 (0.10–0.64)	0.17 (0.08–0.65)	0.23 (0.05–1.33)	0.12 (0.04–0.58)	0.12 (0.03–0.63)	0.12 (0.00–1.37)	0.28 (0.11–1.16)	0.25 (0.08–1.17)	0.40 (0.05–2.88)
Sjogren's syndrome	0.32 (0.20–0.98)	0.36 (0.22–1.14)	0.15 (0.02–1.10)	0.07 (0.02–0.43)	0.09 (0.02–0.54)	0 (0.00–0.91)	0.72 (0.43–2.28)	0.80 (0.46–2.61)	0.40 (0.05–2.88)
Polymyositis/ dermatomyositis	0.15 (0.07–0.56)	0.15 (0.07–0.60)	0.15 (0.02–1.10)	0.15 (0.05–0.65)	0.16 (0.05–0.72)	0.12 (0.00–1.37)	0.16 (0.04–0.82)	0.15 (0.03–0.88)	0.20 (0.01–2.22)
Behcet's disease	0.72 (0.53–1.91)	0.71 (0.50–1.95)	0.76 (0.36–2.79)	0.54 (0.34–1.65)	0.62 (0.38–1.91)	0.25 (0.03–1.77)	1.00 (0.65–2.96)	0.85 (0.50–2.73)	1.59 (0.69–6.28)
ANCA-associated vasculitis	0.03 (0.01–0.22)	0.04 (0.01–0.28)	0.00 (0.00–0.56)	0 (0.00–0.18)	0 (0.00–0.23)	0 (0.00–0.91)	0.08 (0.01–0.58)	0.10 (0.01–0.73)	0 (0.00–1.47)
Polyarteritis nodosa	0.03 (0.01–0.22)	0.02 (0.00–0.21)	0.08 (0.00–0.85)	0 (0.00–0.18)	0 (0.00–0.23)	0 (0.00–0.91)	0.08 (0.01–0.58)	0.05 (0.00–0.56)	0.20 (0.01–2.22)
Takayasu's arteritis	0.09 (0.03–0.40)	0.12 (0.04–0.50)	0.00 (0.00–0.56)	0.07 (0.02–0.43)	0.09 (0.02–0.54)	0 (0.00–0.91)	0.12 (0.03–0.70)	0.15 (0.03–0.88)	0 (0.00–1.47)
Adult-onset Still's disease	0.31 (0.19–0.95)	0.27 (0.15–0.90)	0.46 (0.17–1.98)	0.22 (0.10–0.85)	0.19 (0.07–0.81)	0.37 (0.08–2.15)	0.44 (0.22–1.58)	0.40 (0.17–1.58)	0.60 (0.12–3.49)
Juvenile idiopathic arthritis	2.31 (1.96–5.42)	2.03 (1.66–4.91)	3.42 (2.49–9.15)	2.25 (1.81–5.53)	1.95 (1.50–4.99)	3.44 (2.28–9.93)	2.41 (1.84–6.20)	2.16 (1.56–5.82)	3.39 (1.97–10.85)
Juvenile ankylosing spondylitis	0.38 (0.25–1.13)	0.36 (0.22–1.14)	0.46 (0.17–1.98)	0.52 (0.32–1.59)	0.46 (0.26–1.53)	0.74 (0.27–3.21)	0.16 (0.04–0.82)	0.20 (0.06–1.03)	0 (0.00–1.47)
Systemic juvenile idiopathic arthritis	0.12 (0.05–0.48)	0.10 (0.03–0.45)	0.23 (0.05–1.33)	0.15 (0.05–0.65)	0.09 (0.02–0.54)	0.37 (0.08–2.15)	0.08 (0.01–0.58)	0.1 (0.01–0.73)	0 (0.00–1.47)
Seronegative juvenile idiopathic arthritis	0.23 (0.13–0.76)	0.25 (0.13–0.85)	0.15 (0.02–1.10)	0.20 (0.09–0.78)	0.22 (0.09–0.89)	0.12 (0.00–1.37)	0.28 (0.11–1.16)	0.30 (0.11–1.31)	0.20 (0.01–2.22)
Overlap syndrome	0.08 (0.03–0.36)	0.08 (0.02–0.39)	0.08 (0.01–0.85)	0 (0.00–0.18)	0 (0.00–0.23)	0 (0.00–0.91)	0.2 (0.07–0.94)	0.20 (0.06–1.03)	0.20 (0.01–2.22)
Relapsing polychondritis	0.05 (0.01–0.27)	0.06 (0.01–0.34)	0.00 (0.00–0.56)	0.05 (0.01–0.36)	0.06 (0.01–0.45)	0 (0.00–0.91)	0.04 (0.00–0.45)	0.05 (0.00–0.56)	0 (0.00–1.47)
Crohn's disease	4.59 (4.09–10.28)	4.43 (3.87–10.07)	5.24 (4.08–13.27)	5.22 (4.54–11.94)	5.11 (4.36–11.90)	5.65 (4.13–15.06)	3.57 (2.86–8.78)	3.31 (2.56–8.43)	4.58 (2.91–13.75)
Ulcerative colitis	5.78 (5.21–12.80)	6.00 (5.35–13.40)	4.94 (3.81–12.58)	6.87 (6.09–15.46)	7.09 (6.20–16.14)	6.01 (4.45–15.90)	4.01 (3.26–9.76)	4.22 (3.36–10.44)	3.19 (1.82–10.35)
Diabetes mellitus, type 1	11.62 (10.81–24.95)	10.87 (10.00–23.60)	14.60 (12.60–33.62)	11.18 (10.18–24.53)	10.29 (9.21–22.91)	14.74 (12.22–35.24)	12.32 (10.98–27.56)	11.81 (10.35–26.84)	14.36 (11.23–36.16)
Hashimoto's thyroiditis	19.31 (18.26–40.80)	17.89 (16.76–38.14)	24.94 (22.31–55.58)	7.67 (6.84–17.14)	7.09 (6.20–16.15)	9.94 (7.90–24.72)	38.22 (35.83–81.46)	35.42 (32.85–76.27)	49.34 (43.38–111.79)
Graves' disease	20.81 (19.72–43.89)	19.29 (18.11–41.03)	26.84 (24.11–59.58)	10.51 (9.54–23.12)	9.88 (8.83–22.05)	13.01 (10.66–31.48)	37.53 (35.16–80.03)	34.56 (32.02–74.48)	49.33 (43.37–111.77)
Autoimmune diseases 1^*^	69.54 (67.53–143.18)	65.26 (63.09–135.00)	86.50 (81.54–183.36)	50.67 (48.50–105.83)	48.15 (45.78–101.20)	60.67 (55.43–132.54)	100.24 (96.34–208.52)	93.11 (88.90–194.91)	128.60 (118.83–277.90)
Autoimmune diseases 2^†^	21.66 (20.55–45.64)	20.66 (19.45–43.87)	25.62 (22.96–57.01)	22.99 (21.54–49.04)	22.35 (20.74–48.07)	25.55 (22.20–58.53)	19.506 (17.81–42.64)	17.94 (16.13–39.80)	25.73 (21.48–61.14)

ANCA, antineutrophil cytoplasmic antibody. ^*^The sum of individual diseases, with cases involving two or more diseases counted as one person. ^†^The sum of individual diseases, excluding diabetes mellitus type 1, Hashimoto's disease, and Graves' disease, with cases involving two or more diseases counted as one person.

### HRs for autoimmune diseases according to the surgery

Table 3 lists the HRs for autoimmune diseases according to surgery. Surgery was significantly associated with an increased risk of SLE, AS, JIA, type 1 DM, Hashimoto's thyroiditis, and Graves' disease. Overall, surgery was associated with an increased risk of autoimmune diseases (HR 1.32, 95% CI 1.23–1.41). The association was significant in both males (HR 1.25, 95% CI 1.13–1.38; *p* < 0.001) and females (HR 1.37, 95% CI 1.26–1.50; *p* < 0.001). The increased risk of SLE was observed in both sexes, whereas the associations with AS and JIA were significant only in females and males, respectively.

**Table 3:** Hazard ratios for risk of autoimmune diseases according to surgery (reference: non-surgery group).

Outcome	Overall		Male		Female	
	HR (95% CI)	*p*	HR (95% CI)	*p*	HR (95% CI)	*p*
Rheumatoid arthritis	1.15 (0.52–2.53)	0.727	0.99 (0.21–4.67)	0.992	1.22 (0.49–3.06)	0.672
Systemic lupus erythematosus	2.20 (1.55–3.13)	< 0.001	2.35 (1.11–4.97)	0.026	2.17 (1.46–3.22)	< 0.001
Ankylosing spondylitis	1.35 (1.06–1.72)	0.013	1.26 (0.97–1.64)	0.091	2.00 (1.11–3.59)	0.021
Systemic sclerosis	1.31 (0.36–4.84)	0.685	0.97 (0.11–8.68)	0.978	1.59 (0.31–8.19)	0.58
Sjogren's syndrome	0.41 (0.09–1.74)	0.224	0.00 (0.00–Inf)	0.999	0.48 (0.11–2.09)	0.328
Polymyositis/dermatomyositis	1.00 (0.21–4.69)	0.995	0.80 (0.09–6.81)	0.835	1.33 (0.14–12.76)	0.806
Behcet's disease	1.06 (0.53–2.14)	0.866	0.40 (0.09–1.69)	0.210	1.85 (0.80–4.28)	0.153
Adult-onset Still's disease	1.65 (0·63–4.29)	0.306	1.89 (0.47–7.57)	0.368	1.47 (0.39–5.52)	0.573
Juvenile idiopathic arthritis	1.69 (1.19–2.39)	0.003	1.77 (1.13–2.76)	0.012	1.57 (0.90–2.76)	0.113
Juvenile ankylosing spondylitis	1.23 (0.49–3.09)	0.654	1.56 (0.60–4.02)	0.360	0.00 (0.00–Inf)	0.998
Systemic juvenile idiopathic arthritis	2.37 (0.57–9.92)	0.237	3.94 (0.79–19.50)	0.093	0.00 (0.00–Inf)	0.999
Seronegative juvenile idiopathic arthritis	0.61 (0.14–2.71)	0.518	0.57 (0.07–4.62)	0.597	0.66 (0.08–5.52)	0.705
Crohn's disease	1.18 (0.90–1.55)	0.223	1.1 (0.79–1.53)	0.568	1.39 (0.86–2.23)	0.176
Ulcerative colitis	0.80 (0.62–1.05)	0.108	0.83 (0.61–1.12)	0.221	0.74 (0.43–1.27)	0.272
Diabetes mellitus, type 1	1.34 (1.14–1.58)	< 0.001	1.43 (1.16–1.76)	< 0.001	1.22 (0.94–1.59)	0.144
Hashimoto's thyroiditis	1.39 (1.22–1.57)	< 0.001	1.39 (1.08–1.79)	0.011	1.39 (1.20–1.60)	< 0.001
Graves' disease	1.38 (1.22–1.56)	< 0.001	1.31 (1.05–1.63)	0.017	1.42 (1.23–1.64)	< 0.001
Autoimmune diseases 1^*^	1.32 (1.23–1.41)	< 0.001	1.25 (1.13–1.38)	< 0.001	1.37 (1.26–1.50)	< 0.001
Autoimmune diseases 2^†^	1.22 (1.08–1.38)	0.001	1.12 (0.96–1.31)	0.137	1.42 (1.16–1.74)	< 0.001

HR, hazard ratio. Inf, infinity. ^*^The sum of individual diseases, with cases involving two or more diseases counted as one person. ^†^The sum of individual diseases, excluding diabetes mellitus type 1, Hashimoto's disease, and Graves' disease, with cases involving two or more diseases counted as one person. ANCA-associated vasculitis, polyarteritis nodosa, Takayasu arteritis, relapsing polychondritis, and overlap syndrome were excluded because the number of events was insufficient for reliable hazard ratio estimation.

The median times from the index date to the development of autoimmune disease were 3,930 days in the non-surgery group and 4,079 days in the surgery group, with a median difference of −148 days (*p* < 0.001).

### Cumulative incidence curves of autoimmune diseases

**Figure 2:**
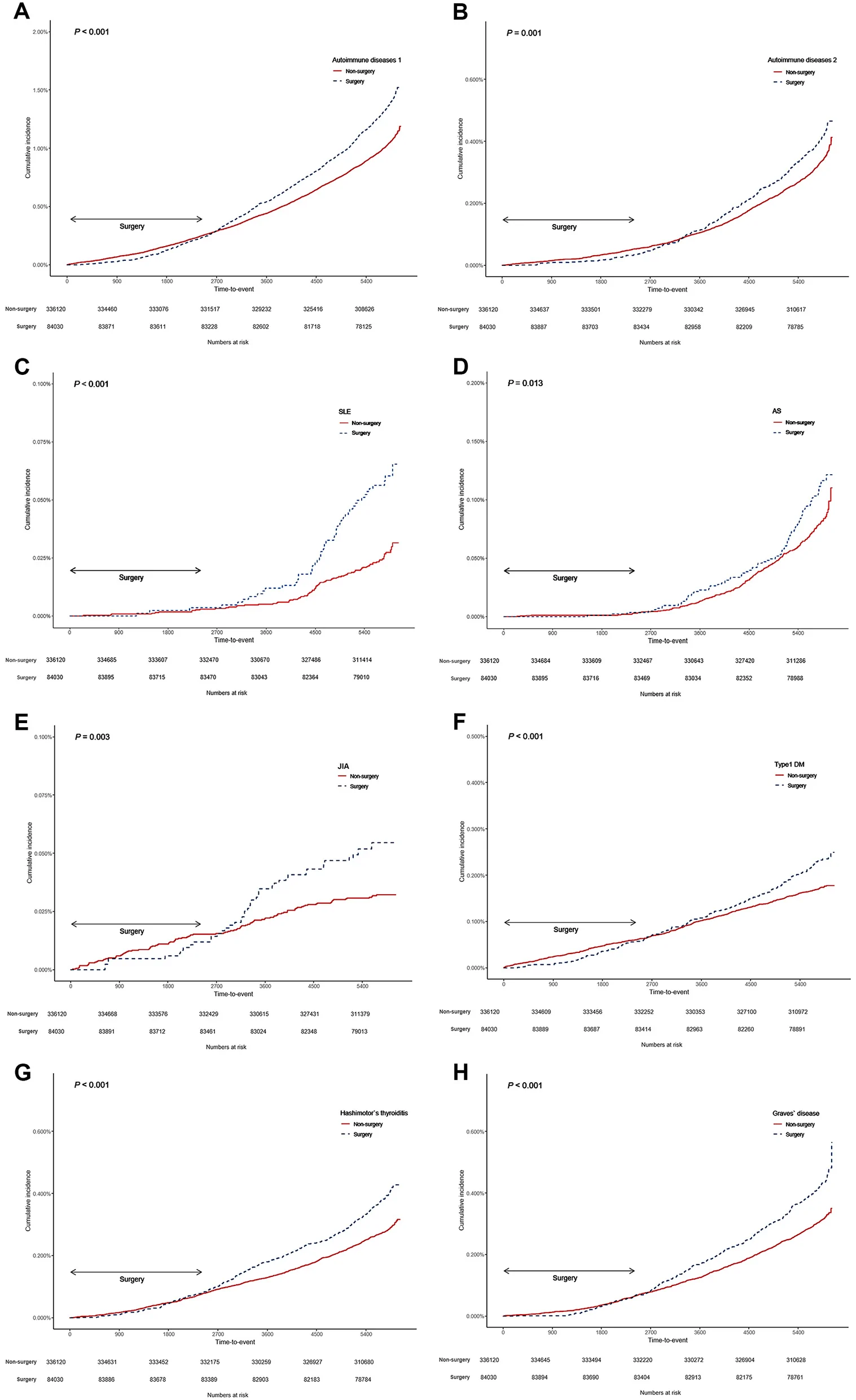
Cumulative incidence curve of autoimmune diseases in surgery and non-surgery groups. Cumulative incidence curves comparing the risk of autoimmune diseases between the surgery and non-surgery groups over time. The y-axis represents the cumulative incidence and the x-axis represents time-to-event in days. The period indicated by the additional arrow represents the time during which surgery was performed in the surgery group. **(A)** Overall incidence of autoimmune diseases. **(B)** Cumulative incidence of autoimmune disease 2. **(C)** Cumulative incidence of SLE. **(D)** Cumulative incidence of AS. **(E)** Cumulative incidence of JIA. **(F)** Cumulative incidence of type 1 DM. **(G)** Cumulative incidence of Hashimoto's thyroiditis. **(H)** Cumulative incidence of Graves' disease. Autoimmune disease 1 refers to the total number of individual diseases, with cases involving two or more diseases counted as one person. Autoimmune disease 2 refers to the total number of individual diseases, excluding type 1 DM, Hashimoto's thyroiditis, and Graves' disease, with cases involving two or more diseases counted as one person. SLE, systemic lupus erythematosus; AS, ankylosing spondylitis; JIA, juvenile idiopathic arthritis; DM, diabetes mellitus.

Figure 2 shows the cumulative incidence curves of autoimmune diseases in the surgery and non-surgery groups during the follow-up period. The x-axis starts from the index date, with the period during which surgery was performed in the surgery group marked separately with an arrow. After the surgery period, the surgery group had a significantly higher incidence of autoimmune diseases compared with the non-surgery group (Figures 2A, B). This trend was consistent for each disease, including SLE (Figure 2C), AS (Figure 2D), JIA (Figure 2E), type 1 DM (Figure 2F), Hashimoto's thyroiditis (Figure 2G), and Graves' disease (Figure 2H).

### Subgroup analysis within the surgery group

Subgroup analysis was performed within the surgery group. In the surgery group (*n* = 84,030), 52,153 patients were male (62.0%) and 31,877 were female (38.0%). The mean age at the time of surgery was 7.60 years (SD 3.04) overall; 7.49 years (SD 3.03) for males and 7.82 years (SD 3.04) for females. Out of a total of 84,030 children, 5,755 underwent adenoidectomy (6.9%), 9,682 underwent tonsillectomy (11.5%), and 68,593 underwent both adenoidectomy and tonsillectomy (81.6%). Regarding the diagnosis at the time of surgery, 39,900 children (47.5%) were classified as inflammation, and 44,130 children (52.5%) were classified as non-inflammation. HRs for autoimmune diseases were analyzed by sex, surgery type, and reason for surgery (Supplementary Table 3). Females were at increased risk of developing autoimmune diseases compared with males (HR 2.11, 95% CI 1.88–2.38). Compared with adenoidectomy, tonsillectomy was associated with an increased risk of autoimmune disease development (HR 1.35, 95% CI 1.01–1.81). Regarding the reason for surgery (inflammatory vs non-inflammatory disease), there was no significant difference (HR 0.91, 95% CI 0.81–1.03). In the surgery group, the median time from surgery to autoimmune disease development was 3,326 days. When analyzed by sex, the median time from surgery to autoimmune disease development was 3,326 days in males and 3,106 days in females, with a median difference of 220 days (*p* = 0.001), indicating that females developed autoimmune diseases slightly earlier than did males.

## Discussion

Our nationwide population-based study found that tonsillectomy and/or adenoidectomy is associated with an increased risk of developing several autoimmune diseases. We evaluated 22 autoimmune diseases and found that surgery was associated with an increased risk of SLE, AS, JIA, type 1 DM, Hashimoto's thyroiditis, and Graves' disease. These findings are generally consistent with those observed in a population-based cohort study conducted in Sweden (13).

The strengths of the present study include its extensive, unique large-scale study population in Asia and the use of a long-term follow-up period in children who underwent tonsillectomy and/or adenoidectomy. Additionally, we conducted an analysis based on sex. The accuracy of the diagnoses is assured, as autoimmune diseases classified under the RID designation system are diagnosed through a comprehensive evaluation by the treating physician, adhering to the standardized diagnostic criteria established by the NHIS in South Korea. (14) The precision of disease diagnoses and the comprehensive national assessment of autoimmune disease incidence following tonsillectomy and/or adenoidectomy are critical components of this study.

We focused exclusively on children aged under 12 years, although our study had a longer average follow-up period (15.5 years) than the Swedish study, which included 22% of participants aged 20 years or older and had an average follow-up period of 8 years. Our study has limitations in assessing the onset of autoimmune diseases that typically occur in adulthood. Although it would have been beneficial to extend the follow-up period into adulthood, 15.5 years was the maximum follow-up duration available using the current data from the HIRA. For several rheumatic diseases, the small number of cases did not reach statistical significance. Nevertheless, the increase in the incidence of major rheumatic diseases, such as JIA, SLE, and AS, was considered clinically significant. When analyzed by sex, the incidence of autoimmune diseases increased in both males and females. For SLE, the surgery group showed a significant increase in both sexes with similar HRs; AS was significantly higher in females, while JIA increased significantly in males. In subgroup analyses of the surgery group, the incidence of autoimmune diseases was significantly higher in females compared with males, likely due to the generally higher prevalence of autoimmune diseases.

When examining the incidence rates, type 1 DM, Hashimoto's thyroiditis, and Graves' disease showed higher incidences compared with other autoimmune diseases. While the naturally higher incidence of endocrine-related diseases may partly explain this finding (17), these three conditions were not included in the RID system and were defined only by ICD codes. Because this may have led to over-sampling of these three conditions, we conducted an additional analysis that excluded the three endocrine diseases. The results of this analysis were consistent with those obtained when all 22 autoimmune diseases were included.

Tonsillectomy and adenoidectomy are frequently performed in children worldwide (6). The tonsils and adenoids function as secondary lymphoid organs, facilitating antigen processing and the maturation of immune cells to regulate bacterial colonization and infection within the upper respiratory tract (18). They generate diverse B-cell populations and induce secretory IgA responses, thereby contributing to mucosal immune homeostasis and protection against invading pathogens (2, 19). A stepwise program of extrathymic T-cell development reportedly occurs within the human tonsils (20). The presence and persistence of diverse antigen-specific B- and T-cell subtypes in the tonsils and adenoids contribute to immune memory in human upper airways and may play a protective role against pathogens (3). Given that the tonsils and adenoids are lymphoid tissues with immunological functions, it is reasonable to hypothesize that their removal could adversely affect the immune system. However, definitive conclusions have yet to be established, and the current evidence suggests that this has little impact on the immune system. Systematic reviews have found that tonsillectomy has no clinically significant negative effect on the immune system (7). Although the available evidence does suggest these surgeries have no negative impact on humoral immunity, and some conflicting results have been reported, there does not appear to be a significant effect on cellular immunity (21). Patients in the tonsillectomy group showed significantly lower levels of IgM, IgA, and IgG compared with the control group that did not undergo tonsillectomy, 4 to 6 years after the tonsillectomy (22). Adenotonsillectomy in children led to a short-term increase in TCD4+ cell counts and a long-term reduction in IgA and IgG levels, although these remained within normal ranges (23). A study of the long-term impact of tonsillectomy on susceptibility to upper respiratory infections and alterations in humoral and cellular immunity reported minor changes in IgA concentrations and lymphocyte subsets without an associated rise in upper respiratory tract infections (24).

Although surgery does not significantly affect immune function, a notable difference was observed in the incidence of clinical diseases. Byars et al. (25) reported long-term clinical outcomes, rather than immunologic findings, using Danish national registries. In their study of nearly 1.2 million children, the removal of adenoids or tonsils in childhood was associated with a significantly increased relative risk of developing respiratory, allergic, and infectious diseases later in life. A recent study also reported that children who underwent adeno-tonsillectomy had a significantly higher risk of acquiring COVID-19, and potentially other viral upper respiratory tract infections than their siblings (26). A population-based cohort study with a follow-up period of more than 3 years found that tonsillectomy significantly increased the overall risk of developing cancer (27). Regarding autoimmune diseases, Ji et al. (13) investigated a wide range of conditions using national data from Sweden. In their study of 179,875 individuals who underwent tonsillectomy, 5,357 developed autoimmune diseases. The standardized incidence ratio was 1.34 compared with the general population. The study evaluated 33 autoimmune diseases, with the incidence of 16 showing a significant increase, and no diseases showing a decreased incidence.

While the precise mechanisms linking tonsillectomy/adenoidectomy to later systemic autoimmune or immune-mediated inflammatory diseases remain unclear, several potential pathways may be considered. Recurrent preoperative streptococcal tonsillar infections may contribute to systemic immune dysregulation through repeated antigenic stimulation or molecular mimicry, potentially triggering autoimmune responses in genetically susceptible individuals (28). In this context, tonsillectomy may partly represent a clinical marker of recurrent infection burden or shared immune susceptibility rather than acting as a direct causal factor. Another possible explanation is that removal of tonsillar/adenoidal lymphoid tissue alters local mucosal immune surveillance and host–microbial immune regulation in the upper airway. Several studies have reported that the composition of the oropharyngeal and gut microbiota changes after tonsillectomy (29, 30). These alterations may further influence host–microbial interactions and contribute to immune dysregulation. Furthermore, Waldeyer's ring-associated lymphoid tissues may play an important role in the induction of immune tolerance during early childhood by promoting appropriate immune responses to self-antigens and commensal microorganisms (3, 31). Early removal of these lymphoid tissues may interfere with this process, potentially predisposing susceptible individuals to dysregulated immune responses and subsequent autoimmune disease

These mechanisms may also contribute to disease-specific pathways. A study of a population-based cohort in Taiwan found that pediatric tonsillectomy increased the risk of developing aggressive and acute periodontitis 4 years or more after surgery (32). Periodontitis is a well-known environmental risk factor for developing RA (33). Both the immunological role of the tonsils and the increased incidence of periodontitis due to tonsillectomy may contribute to the development of RA. Aside from the Swedish study, to the best of our knowledge, no studies examining the relationship between tonsillectomy and AS or SLE have been conducted. We found that the incidence of type 1 DM increased after tonsillectomy or adenoidectomy. However, a previous study of 25,488 children in Northern Ireland reported finding no increased risk of childhood-onset type 1 DM after tonsillectomy or adenoidectomy (34). In contrast, a Swedish study observed an increased incidence of type 1 DM, Graves' disease, and Hashimoto's thyroiditis in a tonsillectomy group (13). These conflicting findings suggest potential regional or population-specific differences, highlighting the need for further investigation.

The finding that tonsillectomy was associated with a higher risk of autoimmune disease than adenoidectomy alone may also be mechanistically informative. Although palatine tonsils and adenoids are both components of Waldeyer's ring, they differ in anatomical location, antigen exposure, epithelial structure, and immune-cell composition (35). The palatine tonsils are directly exposed to the oropharyngeal environment and contain deep crypt epithelium, which may facilitate sustained interaction with bacterial antigens and contribute to local immune surveillance, mucosal antibody responses, and host–microbial immune regulation (36). In addition, bilateral palatine tonsillectomy may result in a greater reduction of oropharyngeal lymphoid tissue burden than adenoidectomy alone, potentially leading to broader alterations in local mucosal immune surveillance. However, tissue volume was not measured in this claims-based study, and surgery type was not randomly assigned. Therefore, this subgroup finding should be interpreted cautiously as hypothesis-generating.

This study has several limitations. The decision to perform tonsillectomy and/or adenoidectomy may itself reflect more severe or recurrent tonsillar/adenoid disease, greater infection burden, upper airway obstruction, or other unmeasured clinical characteristics associated with later development of systemic autoimmune or immune-mediated inflammatory diseases. Furthermore, detailed clinical information regarding disease severity, such as the frequency of tonsillitis episodes, disease duration before surgery, and preoperative immunological characteristics, was not available in the HIRA database, which may have resulted in residual confounding related to disease severity. Although age and sex were matched and patients with malignancy and brain tumors were excluded, the potential influence of other factors associated with autoimmune disease development, including socioeconomic status, region, obesity, recurrent infections, family history of autoimmune diseases, and allergic conditions such as atopy and asthma, could not be fully excluded because these variables were not adequately captured in the HIRA dataset. In addition, Periodic fever, aphthous stomatitis, pharyngitis, and cervical adenitis (PFAPA) syndrome was not specifically identified or excluded, and its potential influence on the study findings cannot be excluded. In contrast with a Swedish study, which compared the incidence of autoimmune diseases between the general population and the tonsillectomy cohort, this study mitigated the potential confounding factors by selecting a control group with identical underlying conditions, such as tonsil or adenoid diseases, through an age- and sex-matched design. This approach enhanced the validity of the findings by minimizing the impact of baseline disease-related differences between the study groups. Additionally, because tonsillectomy/adenoidectomy was performed after the index date, the possibility of immortal time bias cannot be completely excluded. However, the index date was defined as the first diagnosis date of tonsillar or adenoid disease in both groups to ensure comparable baseline characteristics. Information on mortality and emigration was not available in the HIRA database. Therefore, competing risks related to these events could not be evaluated. Finally, because multiple autoimmune diseases and subgroup analyses were evaluated, adjustment for multiple comparisons was not performed, and the findings should be interpreted with caution. Although several associations were statistically significant, the absolute incidence of autoimmune diseases was relatively low. Therefore, the observed associations should be interpreted in the context of both relative and absolute risks.

Despite these limitations, the results of the present study, which involved a unique and large-scale investigation in Asia, provide important insights for future research on the association between tonsillectomy and/or adenoidectomy and the development of autoimmune diseases.

In conclusion, tonsillectomy and/or adenoidectomy during childhood was associated with an increased risk of autoimmune diseases later in life. These findings suggest a potential association between these procedures and long-term autoimmune disease risk. This association should be interpreted with caution, as it may be influenced by residual confounding or other unmeasured factors inherent to observational studies.

## Data Availability

The data analyzed in this study is subject to the following licenses/restrictions: The datasets used in this study are not publicly available due to legal restrictions governing the Health Insurance Review and Assessment Service (HIRA) claims database. Access is subject to approval by HIRA. Requests to access these datasets should be directed to HIRA Big Data Opening Portal (https://opendata.hira.or.kr) following institutional approval.
