# Supplementary material for: Association between childhood tonsillectomy and increased risk of autoimmune disease: a nationwide population-based cohort study
Source: Front Med (Lausanne). 2026 Aug 11;13:1892240. doi: 10.3389/fmed.2026.1892240 (PMC13503185; doi:10.3389/fmed.2026.1892240)
Supplement: Supplementary file 1 [file Data_Sheet_1.docx]

**Supplementary appendix**

**Supplementary Table 1. ICD-10 and RID codes for autoimmune disease classification**

| **Disease** | **ICD-10** | **RID** |
| --- | --- | --- |
| Rheumatoid arthritis | M058-M059 | V223 |
| Systemic lupus erythematosus | M32, M321, M328, M329 | V136 |
| Ankylosing spondylitis | M45 | V140 |
| Systemic sclerosis | M340, M341, M348, M349 | V138 |
| Sjogren’s syndrome | M350 | V139 |
| Inflammatory myositis |  |  |
| Polymyositis | M332 | V137 |
| Dermatomyositis | M33, M330, M331, M339 | V137 |
| Behcet’s disease | M352 | V139 |
| ANCA-associated vasculitis |  |  |
| Wegener’s granulomatosis | M313 | V135 |
| Microscopic polyangiitis | M317 | V238 |
| Churg-Strauss syndrome | M301 | V135 |
| Polyarteritis nodosa | M300, M302 | V134 |
| Takayasu’s arteritis | M314 | V135 |
| Adult-onset Still’s disease | M061 | V298 |
| Juvenile idiopathic arthritis | M080 | V133 |
| Juvenile ankylosing spondylitis | M081 | V133 |
| Systemic juvenile idiopathic arthritis | M082 | V133 |
| Seronegative juvenile idiopathic arthritis | M083 | V133 |
| Overlap syndrome | M351 | V139 |
| Relapsing polychondritis | M941 | V178 |
| Crohn’s disease | K50 | V130 |
| Ulcerative colitis | K51 | V131 |
| Diabetes mellitus, type 1 | E10 | ·· |
| Hashimoto’s thyroiditis | E063 | ·· |
| Graves’s disease | E050 | ·· |

ICD-10=International Classification of Diseases, Tenth Revision. RID=rare intractable disease.

**Supplementary Table 2. Comparison of the incidence of autoimmune disease between the surgery and non-surgery groups during the follow-up period (2007–2023)**

|  | **All (n=420,150)** | | | | **Male (n=260,765)** | | | **Female (n=159,385)** | | |
| --- | --- | --- | --- | --- | --- | --- | --- | --- | --- | --- |
|  | **Overall**  **(n=420,150)** | **Non-surgery**  **(n=336,120)** | **Surgery**  **(n=84,030)** | **p** | **Non-surgery**  **(n=208,612)** | **Surgery**  **(n=52,153)** | **p** | **Non-surgery**  **(n=127,508)** | **Surgery**  **(n=31,877)** | **p** |
| Rheumatoid arthritis | 35 (0.008%) | 27 (0.008%) | 8 (0.010%) | 0.833 | 8 (0.004%) | 2 (0.004%) | 1.000 | 19 (0.015%) | 6 (0.019%) | 0.803 |
| Systemic lupus erythematosus | 136 (0.032%) | 87 (0.026%) | 49 (0.058%) | <0.001 | 18 (0.009%) | 11 (0.021%) | 0.029 | 69 (0.054%) | 38 (0.119%) | <0.001 |
| Ankylosing spondylitis | 349 (0.083%) | 259 (0.077%) | 90 (0.107%) | 0.008 | 226 (0.108%) | 73 (0.140%) | 0.066 | 33 (0.026%) | 17 (0.053%) | 0.022 |
| Systemic sclerosis | 12 (0.003%) | 9 (0.003%) | 3 (0.004%) | 0.942 | 4 (0.002%) | 1 (0.002%) | 1.000 | 5 (0.004%) | 2 (0.006%) | 0.925 |
| Sjogren’s syndrome | 21 (0.005%) | 19 (0.006%) | 2 (0.002%) | 0.354 | 3 (0.001%) | 0 (0.000%) | 0.885 | 16 (0.013%) | 2 (0.006%) | 0.517 |
| Polymyositis/dermatomyositis | 10 (0.002%) | 8 (0.002%) | 2 (0.002%) | 1.000 | 5 (0.002%) | 1 (0.002%) | 1.000 | 3 (0.002%) | 1 (0.003%) | 1.000 |
| Behcet’s disease | 47 (0.011%) | 37 (0.011%) | 10 (0.012%) | 0.971 | 20 (0.010%) | 2 (0.004%) | 0.311 | 17 (0.013%) | 8 (0.025%) | 0.211 |
| ANCA-associated vasculitis | 2 (0.000%) | 2 (0.001%) | 0 (0.000%) | 1.000 | 0 (0.000%) | 0 (0.000%) | ·· | 2 (0.002%) | 0 (0.000%) | 1.000 |
| Polyarteritis nodosa | 2 (0.000%) | 1 (0.000%) | 1 (0.001%) | 0.860 | 0 (0.000%) | 0 (0.000%) | ·· | 1 (0.001%) | 1 (0.003%) | 0.860 |
| Takayasu’s arteritis | 6 (0.001%) | 6 (0.002%) | 0 (0.000%) | 0.475 | 3 (0.001%) | 0 (0.000%) | 0.885 | 3 (0.002%) | 0 (0.000%) | 0.885 |
| Adult-onset Still’s disease | 20 (0.005%) | 14 (0.004%) | 6 (0.007%) | 0.402 | 6 (0.003%) | 3 (0.006%) | 0.560 | 8 (0.006%) | 3 (0.009%) | 0.821 |
| Juvenile idiopathic arthritis | 151 (0.036%) | 106 (0.032%) | 45 (0.054%) | 0.004 | 63 (0.030%) | 28 (0.054%) | 0.015 | 43 (0.034%) | 17 (0.053%) | 0.146 |
| Juvenile ankylosing spondylitis | 25 (0.006%) | 19 (0.006%) | 6 (0.007%) | 0.803 | 15 (0.007%) | 6 (0.012%) | 0.478 | 4 (0.003%) | 0 (0.000%) | 0.708 |
| Systemic juvenile idiopathic arthritis | 8 (0.002%) | 5 (0.001%) | 3 (0.004%) | 0.426 | 3 (0.001%) | 3 (0.006%) | 0.185 | 2 (0.002%) | 0 (0.000%) | 1.000 |
| Seronegative juvenile idiopathic arthritis | 15 (0.004%) | 13 (0.004%) | 2 (0.002%) | 0.747 | 7 (0.003%) | 1 (0.002%) | 0.930 | 6 (0.005%) | 1 (0.003%) | 1.000 |
| Overlap syndrome | 5 (0.001%) | 4 (0.001%) | 1 (0.001%) | 1.000 | 0 (0.000%) | 0 (0.000%) | ·· | 4 (0.003%) | 1 (0.003%) | 1.000 |
| Relapsing polychondritis | 3 (0.001%) | 3 (0.001%) | 0 (0.000%) | 0.885 | 2 (0.001%) | 0 (0.000%) | 1.000 | 1 (0.001%) | 0 (0.000%) | 1.000 |
| Crohn’s disease | 300 (0.071%) | 231 (0.069%) | 69 (0.082%) | 0.220 | 165 (0.079%) | 46 (0.088%) | 0.570 | 66 (0.052%) | 23 (0.072%) | 0.213 |
| Ulcerative colitis | 378 (0.090%) | 313 (0.093%) | 65 (0.077%) | 0.194 | 229 (0.110%) | 49 (0.094%) | 0.360 | 84 (0.066%) | 16 (0.050%) | 0.381 |
| Diabetes mellitus, type 1 | 759 (0.181%) | 567 (0.169%) | 192 (0.228%) | <0.001 | 332 (0.159%) | 120 (0.230%) | 0.001 | 235 (0.184%) | 72 (0.226%) | 0.149 |
| Hashimoto’s thyroiditis | 1 261 (0.300%) | 933 (0.278%) | 328 (0.390%) | <0.001 | 229 (0.110%) | 81 (0.155%) | 0.009 | 704 (0.552%) | 247 (0.775%) | <0.001 |
| Graves’ disease | 1 359 (0.323%) | 1 006 (0.299%) | 353 (0.420%) | <0.001 | 319 (0.153%) | 106 (0.203%) | 0.013 | 687 (0.539%) | 247 (0.775%) | <0.001 |
| Autoimmune diseases 1* | 4 529 (1.078%) | 3 395 (1.010%) | 1 134 (1.350%) | <0.001 | 1 551 (0.743%) | 493 (0.945%) | <0.001 | 1 844 (1.446%) | 641 (2.011%) | <0.001 |
| Autoimmune diseases 2† | 1 415 (0.337%) | 1 078 (0.321%) | 337 (0.401%) | <0.001 | 721 (0.346%) | 208 (0.399%) | 0.075 | 357 (0.280%) | 129 (0.405%) | <0.001 |

Data are n (%). ANCA=antineutrophil cytoplasmic antibody. *The sum of individual diseases, with cases involving two or more diseases counted as one person. †The sum of individual diseases, excluding diabetes mellitus type 1, Hashimoto’s disease, and Graves’ disease, with cases involving two or more diseases counted as one person.

**Supplementary Table 3. Hazard ratios for autoimmune disease by sex, surgery type, and reason for surgery among the surgery group**

| **Outcome** | **Sex (reference: male)** | | **Surgery type (reference: adenoidectomy)** | | | | **Reason for surgery (reference: non-inflammatory*)** | |
| --- | --- | --- | --- | --- | --- | --- | --- | --- |
|  | **Female** | | **Both adenoidectomy plus tonsillectomy** | | **Tonsillectomy** | | **Inflammation†** | |
|  | **HR** | **p** | **HR** | **p** | **HR** | **p** | **HR** | **p** |
| Autoimmune diseases 1 | 2.11 (1.88–2.38) | <0.001 | 1.13 (0.88–1.45) | 0.341 | 1.35 (1.01–1.81) | 0.043 | 0.91 (0.81–1.03) | 0.133 |
| Autoimmune diseases 2 | 1.00 (0.80–1.24) | 0.988 | 1.10 (0.70–1.72) | 0.696 | 1.15 (0.67–1.97) | 0.624 | 0.93 (0.75–1.16) | 0.531 |

HR=hazard ratio. *Non-inflammatory includes enlargement or other chronic changes to the tonsil and adenoid (hypertrophy of tonsil, hypertrophy of adenoid, other chronic disease of tonsil and adenoid). †Inflammation covers acute and chronic infections of abscess of tonsil or adenoid.
